# Supplementary material for: Using cancer risk algorithms to improve risk estimates and referral decisions
Source: Commun Med (Lond). 2022 Jan 10;2:2. doi: 10.1038/s43856-021-00069-1 (PMC9053195; doi:10.1038/s43856-021-00069-1)
Supplement: Supplementary file 3 — Supplemental material [file 43856_2021_69_MOESM3_ESM.pdf]

SUPPLEMENTARY INFORMATION

Using cancer risk algorithms to improve risk estimates and referral decisions

Authors: Olga Kostopoulou*, Kavleen Arora, Bence Pálfi

Affiliation for all authors: Imperial College London, Department of Surgery & Cancer, London, UK

* Corresponding author: Olga Kostopoulou
E-mail: o.kostopoulou@imperial.ac.uk
ORCID iD: https://orcid.org/0000-0001-9643-0838

Supplementary Methods……………………………………………………………2
Supplementary Note 1: Regression tables…………………………………………7
Supplementary Note 2: GP comments……………………………………………..11

Supplementary Methods

1. Practice vignettes

Patient name: Bryony Barnes (female)
Age: 56
BMI: 30
Smoking: Currently smokes 15 cigarettes/day
Alcohol intake: 21 units/week
Bryony Barnes comes to see you complaining of being more constipated in the last month. Over the last 2 months, she has also noted that she has lost about 4kg in weight and doesn't understand why. She has not been dieting and her lifestyle has not changed.
QCANCER RISK: 1.04

Patient name: Henry Lipp (male)
Age: 75
BMI: 24.9
Smoking: Never smoked
Alcohol intake: 4 units/week
Mr Henry Lipp has come to see you concerned because he noticed some blood in his stools over the last four weeks. He has no other symptoms.
QCANCER RISK: 6.33

Patient name: Dawn Jenkins (female)
Age: 70
BMI: 26.2
Smoking: Ex-smoker
Alcohol intake: 14 units/week
Dawn Jenkins is your next patient. She has a background of Type 2 Diabetes and no other medical problems. She has come to see you because in the last few weeks she has become increasingly aware of some right sided abdominal pain. When you ask her about her bowels, she says she has seen some blood on and off when she looks in the toilet and this has been the case for the last two weeks. You ask her to do some blood tests which reveal a microcytic anaemia (Hb 9.8) and a low ferritin.
QCANCER RISK: 39.60

2. Vignettes used for data collection

Patient name: Patricia Staunton (female)
Age: 47
BMI: 26.4
Smoking: Ex-smoker
Alcohol intake: 14 units/week
Patricia Staunton comes to see you accompanied by her husband. Both he and her sister told her to come and see you because she has lost some weight recently. When you ask her how much, she is not sure but thinks it is probably around 3kg in the last 2 months. She says that her clothes definitely feel more loose. She also mentions some 'tummy pain' that she has started to notice in the last month.
QCANCER RISK: 0.58

Patient name: Adam Harper (male)
Age: 57
BMI: 24.6
Smoking: Never smoked
Alcohol intake: 21 units/week
Adam Harper is new to the practice. He comes to see you because his bowels are 'acting up'. On further questioning, he says that in the last few weeks his motions tend to be loose and he opens his

bowels more frequently. He says that he has always been very regular, going once a day, 'like clockwork'. He denies any change in his diet and has no other symptoms.
QCANCER RISK: 0.69

Patient name: Polly Penkin (female)
Age: 52
BMI: 22.4
Smoking: Currently smokes 20 cigarettes/day
Alcohol intake: nil
Polly Penkin comes to see you about some blood that she noticed in her motions in the past month. She says that it seems to be mixed in with the stools. She has no other symptoms but is worried. Looking at her record, you see that she has no medical conditions.
QCANCER RISK: 0.96

Patient name: Matt Crayton (male)
Age: 75
BMI: 24.4
Smoking: Never smoked
Alcohol intake: 4 units/week
Matt Crayton comes in to see you with his wife. He is seeking your advice because he has lost some weight recently. His wife intervenes and says that she and his friends have this over the last 3 months and told him to see the GP. Matt says that he has not changed his diet and has no other symptoms.
QCANCER RISK: 1.08

Patient name: Donna Draper (female)
Age: 62
BMI: 27.2
Smoking: Never smoked
Alcohol intake: 7 units/week
Donna Draper is your next patient. You remember that she came in with her grandson the other day when he was unwell. Today, she comes in to get the results of a routine blood test that she had done recently. It shows microcytic anaemia (Hb 10.4) with low ferritin. She also wants to discuss a new problem with you today: she says that since last month, her stools have become runnier and she is going more frequently during the day. She has not changed her diet and has no other symptoms.
QCANCER RISK: 1.40

Patient name: Dennis Wareham (male)
Age: 80
BMI: 23.6
Smoking: Never smoked
Alcohol intake: Nil
Dennis Wareham, a nice 80-year-old gentleman, came to see you last week complaining of general fatigue over the last 2 months. On further questioning, he denied any other symptoms or weight loss. You decided to organise some routine blood tests. His results came back today, showing microcytic anaemia (Hb 10.0) with low ferritin, which hasn’t been noted before.
QCANCER RISK: 1.52

Patient name: Nina Durbridge (female)
Age: 54
BMI: 27.2
Smoking: Ex-smoker
Alcohol intake: 21 units/week
Nina Durbridge, your next patient, works in marketing. She saw another doctor in your surgery last week and had requested to have some routine blood tests. She was called to come in for the results and has made an appointment for today. The results show microcytic anaemia (Hb 10.8) with a low ferritin. Upon enquiring about any symptoms that she may have, she tells you that she has had lower abdominal pain for about a month, which she has not had before. She has no other symptoms.
QCANCER RISK: 2.09

Patient name: Jack Barton (male)
Age: 45
BMI: 24.6
Smoking: Ex-smoker
Alcohol intake: 21 units/week
Jack Barton comes to see you because he is concerned about some blood that he noticed when he goes to the toilet in the last few weeks. He is seeing it most days, mixed in with his stools. He feels that he has lost some weight, though he is not sure how much. His wife has also noticed it and asked him to mention it to you. He recalls his weight being around 73kg about 2 months ago. You check it today and it is 69kg.
QCANCER RISK: 2.42

Patient name: Holly Hunt (female)
Age: 76
BMI: 24.3
Smoking: Never smoked
Alcohol intake: 7 units/week
Holly Hunt comes in to see you concerned that the skirts she has had for many years have started to feel loose. She does not normally weigh herself, but her family have told her that she is starting to look a lot thinner. Today, she comes in to get the results of her recent blood test that was requested by the doctor she saw last time. It shows microcytic anaemia (Hb 10.5) with low ferritin.
QCANCER RISK: 3.22

Patient name: Daniel Fox (male)
Age: 59
BMI: 27.1
Smoking: Never smoked
Alcohol intake: 22 units/week
Daniel Fox comes to see you. He lives alone – he used to live with his mother (who needed full-time care), but she died of colorectal cancer a few years ago. He has come to see you today as he has lost some weight recently, which his sister noticed and told him to speak to the GP. He thinks he may have lost about 3kg over the past 2 months and he has not changed his diet or lifestyle. Upon further questioning, Daniel mentions that his stools often appear to be loose and he is passing them more frequently than before. This has been ongoing for the past 6 weeks. He has no other symptoms.
QCANCER RISK: 3.65

Patient name: Debbie Lawrence (female)
Age: 58
BMI: 21.8
Smoking: Currently smokes 3 cigarettes/day
Alcohol intake: 3 units/week
Debbie Lawrence comes in accompanied by her husband. She complains of a 'nagging' lower abdominal pain that she has had for more than a month. She says that she cannot understand what might be causing it and that it is not getting better. She has also noticed that she is passing stool more frequently than usual (2 or 3 times a day) in the last few weeks. You order a blood test, which comes back showing microcytic anaemia (Hb 10.3) with low ferritin.
QCANCER RISK: 4.70

Patient name: Antonio DiMarco (male)
Age: 78
BMI: 24.2
Smoking: Currently smokes 12 cigarettes/day
Alcohol intake: Nil
Antonio DiMarco comes to see you because he has lost weight over the last few months despite no changes in his diet. His wife and friends have remarked on it. Antonio also mentions that he’s been passing stool more frequently than usual, which worries him a little because his father died of gastrointestinal cancer.
QCANCER RISK: 5.16

Patient name: Doris Newman (female)
Age: 75
BMI: 29.4
Smoking: Non-smoker
Alcohol intake: nil
Doris Newman has come to see you concerned about some pain on the right side of her abdomen that she has experienced in the last few weeks. It seems to be there most of the time. She says that she usually loves her food but has lost her appetite lately. She likes to check her weight regularly and has also noticed that it has dropped from 55kg to 52kg in the last month. On further questioning, she reveals that her bowels are opening less regularly in the last few weeks, and she finds it more difficult to pass stool.
QCANCER RISK: 8.78

Patient name: Jane Derry (female)
Age: 55
BMI: 20.6
Smoking: Currently smokes 20 cigarettes/day
Alcohol intake: 8 units/week
Jane Derry is grateful that you can fit her in today. She has come to see you after noticing blood in her stools in the last few weeks. She is particularly concerned because her mother has a history of bowel cancer. You perform some initial blood tests, which reveal microcytic anaemia (Hb 10.1) and low ferritin.
QCANCER RISK: 9.20

Patient name: Norman England (male)
Age: 70
BMI: 26.7
Smoking: Current smoker, 5 cigarettes/day
Alcohol intake: 14 units/week
Norman England has come to see you because he has noticed that his stools have become loose and he has been opening his bowels more frequently over the last four weeks. He usually loves his wife's cooking but doesn't feel like eating anymore. He has lost about 4kg of weight in the last 2 months. He has also noticed that he is getting some abdominal pain on and off. He tells you that his father had bowel cancer when he was of a similar age.
QCANCER RISK: 22.82

Patient name: Doris Fielding (female)
Age: 88
BMI: 23.1
Smoking: Ex-smoker
Alcohol intake: Nil
Doris Fielding comes in to see you today. She is usually well and has no significant past medical problems. She tells you that her family have commented that she appears to have lost quite a bit of weight over the past 6 months. She has noticed that her clothes feel looser. On further questioning, you discover that she has had lower abdominal pain for most days in the last 3 months. Her stools seem to be 'more runny' in the last few weeks. You organise some blood tests, which reveal microcytic anaemia (Hb 10.1) and low ferritin.
QCANCER RISK: 20.76

Patient name: Brian Madden (male)
Age: 64
BMI: 30
Smoking: Currently smokes 10 cigarettes/day
Alcohol intake: 28 units/week
Brian Madden has recently retired. From his records, you can see that other than a history of eczema, he has no other medical problems. Today, he is concerned after noticing that he has lost about 4kg in weight in the last 2 months. He keeps an eye on his weight every few weeks. He does not think that his diet has changed. On further enquiry about his bowels, he says that in the last month, his stools seem looser and he noticed some blood in it on a number of occasions.
QCANCER RISK: 38.90

Patient name: Jane Tarley (female)
Age: 75
BMI: 24.9
Smoking: Currently smokes 20 cigarettes/day
Alcohol intake: Nil
Jane Tarley is your next patient. She has a background of COPD for which she takes inhalers. She has become aware of some right-sided, lower abdominal pain in the last month. She tells you that she is off her food, and thinks that she has lost weight. You weigh her and note that she has lost about 3kg in the last 2 months. Jane also tells you that she has had some blood in her stool most days in the last 2 weeks. She has not had anything like this before. She tells you that her brother was recently diagnosed with bowel cancer.
QCANCER RISK: 40.14

Patient name: Henry Walker (male)
Age: 66
BMI: 37.9
Smoking: Currently smokes 20 cigarettes/day
Alcohol intake: 28 units/week
Henry Walker is your next patient. His wife asked him to book an appointment, when he mentioned blood in his stools over the past few weeks. He says that he normally goes to the toilet once a day 'like clockwork' and is irritated that it is now harder to pass stools and he is less regular. His wife has also noticed that he is looking much thinner than usual. He mentions that his clothes are feeling looser. He says that he usually enjoys his food but not so much these days.
QCANCER RISK: 56.65

Patient name: Judy Hailey (female)
Age: 63
BMI: 23.4
Smoking: Currently smokes 10 cigarettes/day
Alcohol intake: Nil
Judy Hailey likes to keep track of her weight and has noticed a drop of 3kg in the last 2 months. She has come to see you about this and wonders if it is because she has lost her appetite. She is not sure why - nothing has changed in her diet or lifestyle. She tells you about some lower abdominal pain that has been bothering her over the last few weeks. It has been harder for her to open her bowels in the last few months. Occasionally, she sees some blood in her stools, which, she hopes, will go away. She tells you that her father had bowel cancer in his fifties.
QCANCER RISK: 57.23

Supplementary Note 1: Regression tables

1. Changes in risk estimates and referral inclination and their association

**DV: Risk estimate changes**	**b**	**SE**	**z**	**P**	**95% CI**
marginal R^2 = 0, conditional R^2 = 0.31					
Constant	10.23	1.40	7.32	<0.001	7.49, 12.97
**Random-effects parameters**	**Estimate**	**SE**			**95% CI**
GP	18.76	2.82			13.96, 25.20
Vignette	35.92	11.61			19.07, 67.67

Table S1. Empty multilevel linear regression model measuring the extent of change in risk estimates post-algorithm

**DV: Referral inclination changes**	**b**	**SE**	**z**	**P**	**95% CI**
marginal R^2 = 0, conditional R^2 = 0.14					
Constant	0.25	0.03	8.69	<0.001	0.20, 0.31
**Random-effects parameters**	**Estimate**	**SE**			**95% CI**
GP	0.05	0.01			0.03, 0.06
Vignette	0.01	0.00			0.00, 0.02

Table S2. Empty multilevel linear regression model measuring the extent of change in the inclination to refer post-algorithm

**DV: Referral inclination changes**	**b**	**SE**	**z**	**P**	**95% CI**
marginal R^2 = 0.11, conditional R^2 = 0.23					
Risk estimate changes	0.016	0.00	18.08	<0.001	0.01, 0.02
Constant	0.09	0.03	2.94	0.003	0.03, 0.15
**Random-effects parameters**	**Estimate**	**SE**			**95% CI**
GP	0.04	0.01			0.03, 0.05
Vignette	0.01	0.00			0.01, 0.02mel

Table S3a. Multilevel linear regression measuring the association between change in risk estimates and change in the inclination to refer post-algorithm.

**DV: Referral inclination change (binary)**	**OR**	**SE**	**z**	**P**	**95% CI**
marginal R^2 = 0.5, conditional R^2 = 0.24					
Risk estimate changes	1.05	0.00	10.89	<0.001	1.04, 1.05
Constant	0.16	0.03	-11.17	<0.001	0.12, 0.22
**Random-effects parameters**	**OR**	**SE**			**95% CI**
GP	1.04	0.18			0.74, 1.45
Vignette	0.29	0.11			0.13, 0.63

Table S3b. Multilevel logistic regression measuring the association between change in risk estimates and change in referral inclination as a binary variable.

2. Impact of algorithm information and position of intuitive risk estimates

DV: Referral inclination changes	b	SE	z	P	95% CI
marginal R^2 = 0.04, conditional R^2 = 0.19					
Position of intuitive risk estimates					
Underestimation	0.32	0.03	9.51	<0.001	0.26, 0.39
Algorithm information					
Provided	0.04	0.04	1.09	0.276	-0.03, 0.12
Constant	0.16	0.04	4.22	0.000	0.08, 0.23
**Random-effects parameters**	**Estimate**	**SE**			**95% CI**
GP	0.05	0.01			0.03, 0.06
Vignette	0.01	0.01			0.32, 0.35

Table S4a. Multilevel linear regression measuring the impact of algorithm information (provided vs. not provided) on changes in referral inclination, and the association with risk under- vs. over-estimation.

DV: Referral inclination change (binary)	OR	SE	z	P	95% CI
marginal R^2 = 0.03, conditional R^2 = 0.25					
Position of intuitive risk estimates					
Underestimation	2.96	0.48	6.62	<0.001	2.14, 4.08
Algorithm information					
Provided	1.13	0.23	0.58	0.55	0.75, 1.68
Constant	0.19	0.04	-8.59	<0.001	0.13, 0.28
**Random-effects parameters**	**Estimate**	**SE**			**95% CI**
GP	1.22	0.20			0.88, 1.69
Vignette	0.24	0.10			0.11, 0.54

Table S4b. Multilevel logistic regression measuring the impact of algorithm information (provided vs. not provided) on changes in referral inclination as a binary variable, and the association with risk under- vs. over-estimation.

DV: Referral inclination changes	b	SE	z	P	95% CI
marginal R^2 = 0.05, conditional R^2 = 0.20					
Position of intuitive risk estimates					
Underestimation	0.33	0.04	9.20	<0.001	0.26, 0.40
Algorithm information					
Provided	0.04	0.04	0.94	0.349	-0.04, 0.11
GP gender					
Female	-0.06	0.04	-1.36	0.174	-0.14, 0.03
GP experience	0.00	0.00	1.21	0.23	-0.00, 0.01
Decision confidence	-0.10	0.04	-2.41	0.016	-0.19, -0.02
Attitude towards risk calculators	0.02	0.01	1.68	0.093	-0.00, 0.05
Constant	0.32	0.16	2.03	0.043	0.01, 0.63
**Random-effects parameters**	**Estimate**	**SE**			**95% CI**
GP	0.04	0.01			0.03, 0.06
Vignette	0.01	0.00			0.01, 0.02

Table S5. Extension of the multilevel linear regression model reported in Table S4a above, by adding GP demographics (gender and experience), general confidence in assessing possible cancers and general attitude towards cancer risk calculators.

DV: Referral inclination change (binary)	OR	SE	z	P	95% CI
marginal R^2 = 0.04, conditional R^2 = 0.25					
Position of intuitive risk estimates					
Underestimation	1.98	0.21	6.43	<0.001	1.61, 2.43
Algorithm information					
Provided	1.05	0.21	0.26	0.792	0.72, 1.55
GP gender					
Female	0.86	0.18	-0.72	0.473	0.58, 1.29
GP experience	1.01	0.01	1.20	0.231	0.99, 1.03
Decision confidence	0.68	0.14	-1.86	0.062	0.45, 1.02
Attitude towards risk calculators	1.17	0.08	2.43	0.015	1.03, 1.33
Constant	0.25	0.19	-1.86	0.063	0.06, 1.08
**Random-effects parameters**					
GP	1.05	0.18			0.75, 1.47

Table S5b. Extension of the multilevel logistic regression model reported in Table S4b above, by adding GP demographics (gender and experience), general confidence in assessing possible cancers and general attitude towards cancer risk calculators. Only one random-effects parameter has been added because the model did not converge with both GP and vignette as random effects.

3. Algorithm impact on decision appropriateness

DV: Decision appropriateness	OR	SE	z	P	95% CI
marginal R^2 = 0.004, conditional R^2 = 0.44					
Time of decision					
Post-algorithm	1.45	0.10	5.53	<0.001	1.27, 1.65
Constant	2.33	0.94	2.10	0.036	1.06, 5.14
**Random-effects parameters**	**Estimate**	**SE**			**95% CI**
GP	0.23	0.05			0.15, 0.35
Vignette	3.17	1.03			1.67, 5.98

Table S6a. Multilevel logistic regression of decision appropriateness on time of decision (pre- vs. post-algorithm). The ‘uncertain’ responses on the 1-5 scale have been included in the model and categorised as ‘inappropriate’.

DV: Decision appropriateness	OR	SE	z	P	95% CI
marginal R^2 = 0.001, conditional R^2 = 0.48					
Time of decision					
Post-algorithm	1.26	0.11	2.63	0.008	1.06, 1.50
Constant	5.36	2.50	3.59	<0.001	2.15, 13.39
**Random-effects parameters**	**Estimate**	**SE**			**95% CI**
GP	0.46	0.10			0.30, 0.69
Vignette	4.17	1.37			2.20, 7.93

Table S6b. Multilevel logistic regression of decision appropriateness on time of decision (pre- vs. post-algorithm). The ‘uncertain’ responses on the 1-5 scale have been excluded from the calculation.

4. Learning

**DV: |Intuitive estimate – algorithm|**	**b**	**SE**	**z**	**P**	**95% CI**
marginal R^2 = 0.002, conditional R^2 = 0.38					
Study session					
Second	-1.63	0.46	-3.51	<0.001	-2.53, -0.72,
Constant	17.32	1.99	8.69	<0.001	13.41, 21.22
**Random-effects parameters**	**Estimate**	**SE**			**95% CI**
GP	26.23	3.93			19.56, 35.18
Vignette	73.93	23.72			39.42, 138.66

Table S7. Multilevel linear regression of the absolute difference between risk estimates pre-algorithm and QCancer risk scores by study session (first vs. second).

**DV: |Intuitive estimate – algorithm|**	**b**	**SE**	**z**	**P**	**95% CI**
marginal R^2 = 0.002, conditional R^2 = 0.38					
Vignette order	-0.14	0.04	-3.54	<0.001	-0.22, -0.06
Constant	18.00	2.03	8.89	<0.001	14.03, 21.97
**Random-effects parameters**	**Estimate**	**SE**			**95% CI**
GP	26.23	3.93			19.56, 35.18
Vignette	74.07	23.76			39.50, 138.91

Table S8. Multilevel linear regression of the absolute difference between risk estimates pre-algorithm and QCancer risk scores on vignette order (1-20).

Supplementary Note 2: GP comments

Comments made by GPs at the end of the study in relation to reflection and learning from the algorithm

GP 20192: *Really enjoyable and thought provoking, made me question and refine my decision making…I felt an element of learning not just problem solving.*
GP 26347: *I would have liked to have a practice at this first before submitting to get used to the idea of algorithms and the statistics, as this improved during the course of this exercise.*
GP 43463: *Very realistic cases. Definite educational value when going through a list of cases like this.*
GP 74216: *Useful exercise, supporting reflection on one's own assumptions.*
GP 78939: *Good cases, slightly difficult to put a number to symptoms but got easier as I went along.*
GP 95191: *I think this would make an interesting teaching resource.*
GP 78939: *Some of the algorithm results for the cases surprised me in that I wouldn't have had the risk that high, so good learning points to consider…Good cases, slightly difficult to put a number to symptoms but got easier as I went along.*
GP 17080: *Very interesting how the algorithm generally estimated much lower than my personal judgement. My personal estimation of likelihood of cancer also appeared relatively 'blunt' (generally just 'high') compared to the algorithm.*

Specific comments about learning how symptoms contributed to cancer risk:

GP 97506: *That was a really useful way to learn about what factors the algorithm weights. Thank you for the opportunity to take part. Feels like the results of this study will be really clinically relevant for day-to-day practice!*
GP 25029: *I think I underestimate the effect of obesity and alcohol when I assess patients! Interesting to see on these patients my internalised score vary most widely.*
GP 29594: *Interesting experience thanks - will make me note abdominal pain as a symptom more.*
GP 61790: *In some cases, the alcohol intake, smoking status or BMI appear to have a significant impact on the algorithm estimate of probability of colorectal cancer. This is helpful.*
GP 95191: *Seemed age/smoking influenced things quite strongly.*
