# Supplementary material for: Using cancer risk algorithms to improve risk estimates and referral decisions
Source: Commun Med (Lond). 2022 Jan 10;2:2. doi: 10.1038/s43856-021-00069-1 (PMC9053195; doi:10.1038/s43856-021-00069-1)
Supplement: Supplementary file 4 — Description of Additional Supplementary Files [file 43856_2021_69_MOESM4_ESM.pdf]

Description of Additional Supplementary Files

File Name: Supplementary Data 1

Description: Supplementary Data 1 contains all the data supporting the manuscript.

File Name: Supplementary Data 2

Description: Supplementary Data 2 is a subset of Supplementary Data 1, supporting Figure 1.
