# Supplementary material for: Using cancer risk algorithms to improve risk estimates and referral decisions
Source: Commun Med (Lond). 2022 Jan 10;2:2. doi: 10.1038/s43856-021-00069-1 (PMC9053195; doi:10.1038/s43856-021-00069-1)
Supplement: Supplementary file 6 — Reporting Summary [file 43856_2021_69_MOESM6_ESM.pdf]

nature research

Corresponding author(s): Olga Kostopoulou

Last updated by author(s): Nov 23, 2021

Reporting Summary

Nature Research wishes to improve the reproducibility of the work that we publish. This form provides structure for consistency and transparency in reporting. For further information on Nature Research policies, see our Editorial Policies and the Editorial Policy Checklist.

Statistics

For all statistical analyses, confirm that the following items are present in the figure legend, table legend, main text, or Methods section.

n/a	Confirmed	
☐	☒	The exact sample size (*n*) for each experimental group/condition, given as a discrete number and unit of measurement
☐	☒	A statement on whether measurements were taken from distinct samples or whether the same sample was measured repeatedly
☐	☒	The statistical test(s) used AND whether they are one- or two-sided<br>*Only common tests should be described solely by name; describe more complex techniques in the Methods section.*
☐	☒	A description of all covariates tested
☒	☐	A description of any assumptions or corrections, such as tests of normality and adjustment for multiple comparisons
☐	☒	A full description of the statistical parameters including central tendency (e.g. means) or other basic estimates (e.g. regression coefficient) AND variation (e.g. standard deviation) or associated estimates of uncertainty (e.g. confidence intervals)
☐	☒	For null hypothesis testing, the test statistic (e.g. *F*, *t*, *r*) with confidence intervals, effect sizes, degrees of freedom and *P* value noted<br>*Give P values as exact values whenever suitable.*
☒	☐	For Bayesian analysis, information on the choice of priors and Markov chain Monte Carlo settings
☐	☒	For hierarchical and complex designs, identification of the appropriate level for tests and full reporting of outcomes
☐	☒	Estimates of effect sizes (e.g. Cohen's *d*, Pearson's *r*), indicating how they were calculated

Our web collection on statistics for biologists contains articles on many of the points above.

Software and code

Policy information about availability of computer code

Data collection	The data were collected on the Qualtrics platform. No computer code or algorithm was used for data collection.
Data analysis	Stata 17.0 and R (version 4.0.3) were used to analyse the data. The standard commands for multilevel regression with random intercepts (crossed random effects) were used. In STATA, the command is: mixed OUTCOME PREDICTOR || _all: R.PARTICIPANT_ID || VIGNETTE_ID: In R, the command is model_full <- lmer(OUTCOME ~ PREDICTOR + (1|PARTICIPANT_ID) + (1|VIGNETTE_ID), data = data)

For manuscripts utilizing custom algorithms or software that are central to the research but not yet described in published literature, software must be made available to editors and reviewers. We strongly encourage code deposition in a community repository (e.g. GitHub). See the Nature Research guidelines for submitting code & software for further information.

Data

Policy information about availability of data

All manuscripts must include a data availability statement. This statement should provide the following information, where applicable:

- Accession codes, unique identifiers, or web links for publicly available datasets
- A list of figures that have associated raw data
- A description of any restrictions on data availability

The dataset has been deposited on Dryad: doi:10.5061/dryad.76hdr7swm. The dataset includes both the raw and calculated data that support Figure 1.

Field-specific reporting

Please select the one below that is the best fit for your research. If you are not sure, read the appropriate sections before making your selection.

☐ Life sciences ☒ Behavioural & social sciences ☐ Ecological, evolutionary & environmental sciences

For a reference copy of the document with all sections, see nature.com/documents/nr-reporting-summary-flat.pdf

Behavioural & social sciences study design

All studies must disclose on these points even when the disclosure is negative.

Study description	Mixed factorial experiment with quantitative data. Some free-text comments are included in the supplementary file to illustrate participants' awareness of learning.
Research sample	157 UK GPs responded to 20 vignettes. The mean age of the GP sample was 44 years (SD 8.7) and 53.5% of the participants were female. We cannot assess sample representativeness, because there are no publicly available age and gender statistics for the UK GP population. It is however known that female GPs (53.5% in our sample) have been outnumbering male GPs since 2014.
Sampling strategy	We recruited a convenience sample of GPs from our database. These were GPs who had participated in previous studies by the lead author. We powered the study to detect a small effect (f2=0.02) of the algorithm on referral decisions with alpha of 5% and power of 95% in a multiple linear regression. The G*Power software (v. 3.1.9.4) estimated that we would need at least 863 responses. To account for data clustering (each GP responding to 20 vignettes), we adjusted this number by the Design Effect (DE). This is calculated using the formula DE=1+(n–1)*ICC, where n is the cluster size (the 20 vignettes), and ICC is the intra-class correlation. We estimated the ICC from pilot data to be 0.088. Thus, DE=2.68. We adjusted the number of participants required by multiplying the 863 required responses with the DE and dividing by the cluster size: (863*2.68)/20=116. Thus, we estimated that we needed to recruit a minimum of 116 GPs.
Data collection	The data were collected entirely online on the Qualtrics platform. GPs could access the study and complete it in their own time.
Timing	Data collection took place between June 27 and September 23, 2020.
Data exclusions	Three respondents were excluded from the calculation of 'experience' (used as a covariate in some of the analyses) because they provided one- or two-digit numbers rather than a year, when asked about the year of their GP qualification.
Non-participation	We invited the 400 GPs in our database of GP e-mail addresses. 157 GPs completed the study, giving a response rate of 39%.
Randomization	Participants were randomly allocated to either receive or not receive information about the study algorithm.

Reporting for specific materials, systems and methods

We require information from authors about some types of materials, experimental systems and methods used in many studies. Here, indicate whether each material, system or method listed is relevant to your study. If you are not sure if a list item applies to your research, read the appropriate section before selecting a response.

Materials & experimental systems

n/a	Involved in the study	
☒	☐	Antibodies
☒	☐	Eukaryotic cell lines
☒	☐	Palaeontology and archaeology
☒	☐	Animals and other organisms
☐	☒	Human research participants
☒	☐	Clinical data
☒	☐	Dual use research of concern

Methods

n/a	Involved in the study	
☒	☐	ChIP-seq
☒	☐	Flow cytometry
☒	☐	MRI-based neuroimaging

Human research participants

Policy information about studies involving human research participants

Population characteristics	Participants were asked to report their age, gender, GP status (fully qualified or GP trainee), year of GP qualification (from which we calculated years of experience), and number of clinical sessions per week.
Recruitment	We sent an invitation email to the 400 GPs in our database of GP e-mail addresses. These are GPs who have participated in other studies by the lead author and have given their permission to be contacted again about studies conducted by our research group. We offered them remuneration of £60 on completion, a completion certificate, and personalised feedback

that they could use for CPD purposes. 157 GPs completed the study. Due to self-selection, it is possible that GPs with an interest in algorithms were more likely to take part, though only 30% of respondents were using cancer risk algorithms in their clinical practice. Thus, it is possible that we measured slightly more positive attitudes towards algorithms than in the general GP population. However, the incentives described above were designed to increase the sample's representativeness by making the study attractive to GPs who did not necessarily have a special interest in algorithmic tools.

Ethics oversight	Study approval was provided by the Health Research Authority (HRA) and Health & Care Research Wales (HCRW), REC reference 20/HRA/2418.

Note that full information on the approval of the study protocol must also be provided in the manuscript.
